# Serial Decline in Lung Volume Parameters on Computed Tomography (CT) Predicts Outcome in Idiopathic Pulmonary Fibrosis (IPF)

**DOI:** 10.1007/s00330-021-08338-2

**Published:** 2021-10-30

**Authors:** Hasti Robbie, Athol U Wells, Cheng Fang, Joseph Jacob, Simon LF Walsh, Arjun Nair, Rose Camoras, Sujal R Desai, Anand Devaraj

**Affiliations:** 1 King's College Hospital NHS Foundation Trust, Denmark Hill, Brixton, London, United Kingdom, SE5 9RS; 2 Royal Brompton and Harefield NHS Foundation Trust, Sydney St, Chelsea, London, United Kingdom, SW3 6NP, National Heart and Lung Institute(NHLI), Dovehouse St, Chelsea, London, United Kingdom, SW3 6LY and Imperial College London, Exhibition Rd, South Kensington, London SW7 2BU; 3 King's College Hospital NHS Foundation Trust, Denmark Hill, Brixton, London, United Kingdom, SE5 9RS; 4 University College London, Gower St, Bloomsbury, London, United Kingdom, WC1E 6BT; 5 National Heart and Lung Institute, Imperial College, London, United Kingdom; 6 University College London Hospital, 235 Euston Rd, Fitzrovia, London, United Kingdom, NW1 2BU; 7 Royal Brompton and Harefield NHS Foundation Trust, Sydney St, Chelsea, London SW3 6NP; 8 Royal Brompton and Harefield NHS Foundation Trust, Sydney St, Chelsea, London, United Kingdom, SW3 6NP; 9 Royal Brompton and Harefield NHS Foundation Trust, Sydney St, Chelsea, London, United Kingdom, SW3 6NP, National Heart and Lung Institute(NHLI), Dovehouse St, Chelsea, London, United Kingdom, SW3 6LY and Imperial College London, Exhibition Rd, South Kensington, London SW7 2BU

**Keywords:** Lung, Tomography, X-Ray Computed, Idiopathic Pulmonary Fibrosis, Disease Progression

## Abstract

**Objectives:**

In patients with IPF: i) to examine the relationship between serial change in CT parameters of lung volume and serial change in lung function, ii) to identify the prognostic value of serial change in CT parameters of lung volume, iii) to define a clinically useful threshold for serial change in CT markers of lung volume that optimally captures disease progression.

**Methods:**

Serial CTs were analysed for evidence of progressive volume loss or fibrosis progression in 81 IPF patients [66 males, median age=67 years] with concurrent Forced Vital Capacity (FVC) measurements (median follow-up 12 months, range 5-23 months). Serial CT measurements of volume loss comprised oblique fissure posterior retraction distance (OFPRD), aortosternal distance (ASD), lung height corrected for body habitus (LH) and automated CT- derived total lung volumes (ALV) (measured using commercially available software). Fibrosis progression was scored visually. Serial changes in CT markers and FVC were compared using regression analysis, and evaluated against mortality using Cox proportional hazards.

**Results:**

There were 58 deaths (72%, median survival=17 months). Annual% change in ALV was most significantly related to annual% change in FVC (R^2^ = 0.26, P<0.0001). On multivariate analysis annual percentage change in ASD predicted mortality (HR=0.97, P<0.001), whereas change in FVC did not. A 25% decline in annual percentage change in ASD best predicted mortality over and above an FVC decline ≥10% and visually assessed fibrosis progression.

**Conclusion:**

In IPF, serial decline in CT markers of regional volume loss and specifically, annualised 25% reduction in aortosternal distance provides evidence of disease progression, not always identified by FVC trends or changes in extent of fibrosis.

## List of Abbreviations

IPF: Idiopathic Pulmonary Fibrosis
PFT: Pulmonary Function Test
TLC: Total Lung Capacity
FVC: Forced Vital Capacity
DLCO: Carbon Monoxide Diffusing Capacity
CPI: Composite Physiological Index
ALV: Automated CT-derived lung volumes
LH: Lung Height
OFPRD: Oblique Fissure Posterior Retraction Distance
ASD: Aortosternal Distance

## Introduction

Idiopathic pulmonary fibrosis (IPF) is characterised by progressive lung parenchymal destruction leading to a gradual decline in respiratory function. Assessing clinical disease progression in IPF is critically important in the management of patients with IPF because it facilitates prognostication and therapeutic decision making, as well as enabling evaluation of treatment response. Disease progression in IPF is usually defined by serial deterioration in pulmonary function tests (PFT), specifically forced vital capacity (FVC). An absolute decline in FVC of 10% is often favoured as an endpoint in many therapeutic trials (1–5). However, serial FVC is poorly sensitive to change (7) and does not always reflect regional morphologic abnormalities in a geographic heterogeneous disease such as IPF.

Several studies have demonstrated that semi-quantitative visual assessment of change in fibrosis extent on serial HRCT can predict poor outcome in IPF (8–11). However, semi-quantitative visual methods of scoring disease progression on CT are subjective and prone to poor interobserver agreement (12). In recent years, there has been growing interest in automated quantitative CT methods of assessing fibrosis extent in IPF (13–21), although the clinical applicability and reproducibility of such methods are yet to be widely established.

Another limitation of both traditional and automated methods of scoring fibrosis extent on CT is that they do not take into account changes in lung volume on CT, which are known to correspond with lung function markers of disease severity in IPF (22). To the best of our knowledge, the evaluation of serial change in lung volume parameters on CT as a marker of disease progression in IPF has also not been previously studied.

Therefore, in this study we aimed to investigate the reliability of serial change in CT parameters of lung volume as a novel marker of disease progression in IPF. To establish clinical validity, we i) evaluated the relationships between serial change in CT parameters of lung volume and FVC, and ii) we aimed to establish the prognostic value of serial change in parameters of volume loss on CT. To establish clinical utility, we iii) aimed to define a clinically applicable threshold for serial change in CT markers of lung volume that optimally predicted outcome.

## Material and Methods

**Figure 1:**
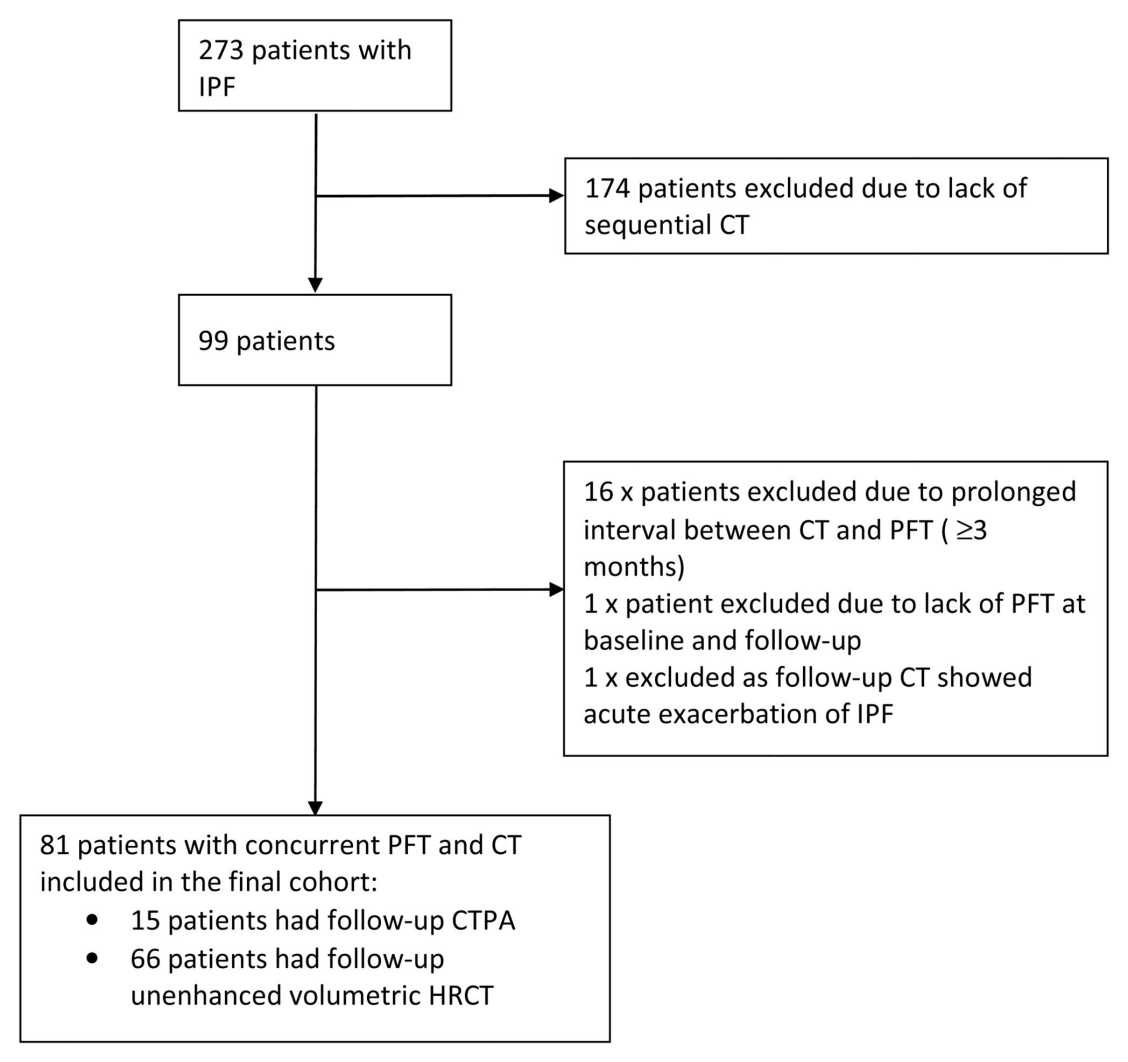
Consort diagram illustrating the process of selecting patients for the final study population.

Institutional review board approval was obtained for this retrospective study and patient consent was not required. We identified 273 consecutive patients with multidisciplinary diagnosis of IPF at our institution between January 2007 and July 2011, of which 99 had follow-up thoracic CT examinations. Seventeen patients were excluded due to lack of concurrent PFTs at both time points (where time between CT and PFT exceeded two months). One patient was excluded due to presence of CT features of acute exacerbation of IPF which could have confounded changes in FVC. The final cohort comprised 81 patients with concurrent PFT and HRCT performed at two time points between January 2007 and 2014 (Figure 1). Median interval between CT and PFTs was 0 months (range 0-2 months). Median follow-up time between baseline and follow up CT studies was 12 months (range 5-23 months). Clinical variables (age and gender) and pulmonary function parameters (FVC, carbon monoxide diffusing capacity (DLCO), and total lung capacity (TLC) expressed in litres and/or percentage predictive values as appropriate) were all acquired from the electronic patient record.

### CT Methods

The CT scans were performed using 64 or 128-slice multiple-detector CT scanners (Siemens, Erlangen, Germany). All scans were reconstructed using a high spatial frequency, B70F kernel. All patients were scanned from lung apices to bases, at full inspiration at peak voltage of 120 kVp with tube current modulation (range, 30 to 140mA), without intravenous contrast at baseline. The standard parameters of 1.4 pitch and 0.5 second rotation time were used. At follow-up, 66 patients (n=66/81) had non-contrast enhanced volumetric CT chest and 15 patients (n=15/81) had CT pulmonary angiogram (CTPA). The CTPA examinations were carried out post injection of 90 ml of intravenous contrast agent (lopromide/Ultravist 300, (300 mg/ml iodine), Bayer Schering, Berlin, Germany) at 4-5 ml/s with Siemens proprietary software bolus tracking used to trigger CT acquisition. Contiguous images of 1 mm thickness were viewed at lung window settings (width l500HU; level - 500HU).

### Quantitative CT-measure of total lung volumes

Syngo CT Pulmo3D package (Pulmo3D version VA30A_HF2, Siemens Medical Solutions, Forchheim, Germany) was used to calculate CT-derived automatic total lung volume (ALV), expressed in litres (L). The software allows automatic segmentation of lungs and lung volume calculation with no requirement for manual editing.

### Surrogate CT measurements of lung volume

**Figure 2:**
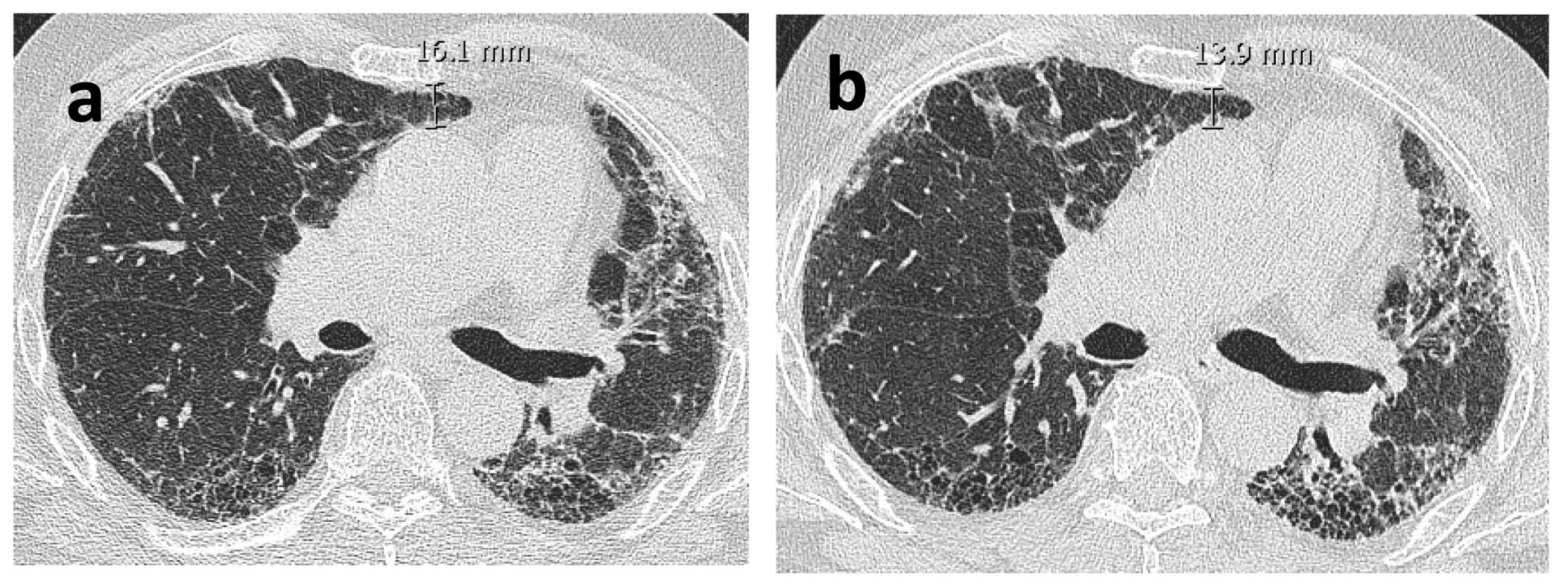
Axial non-contrast enhanced CT images of a 75-year-old male with IPF. Baseline (a) and follow-up (b) images show absence of unequivocal fibrosis progression but a reduction in aortosternal distance, the distance between anterior aspect of ascending thoracic aorta and retrosternum.

**Figure 3:**
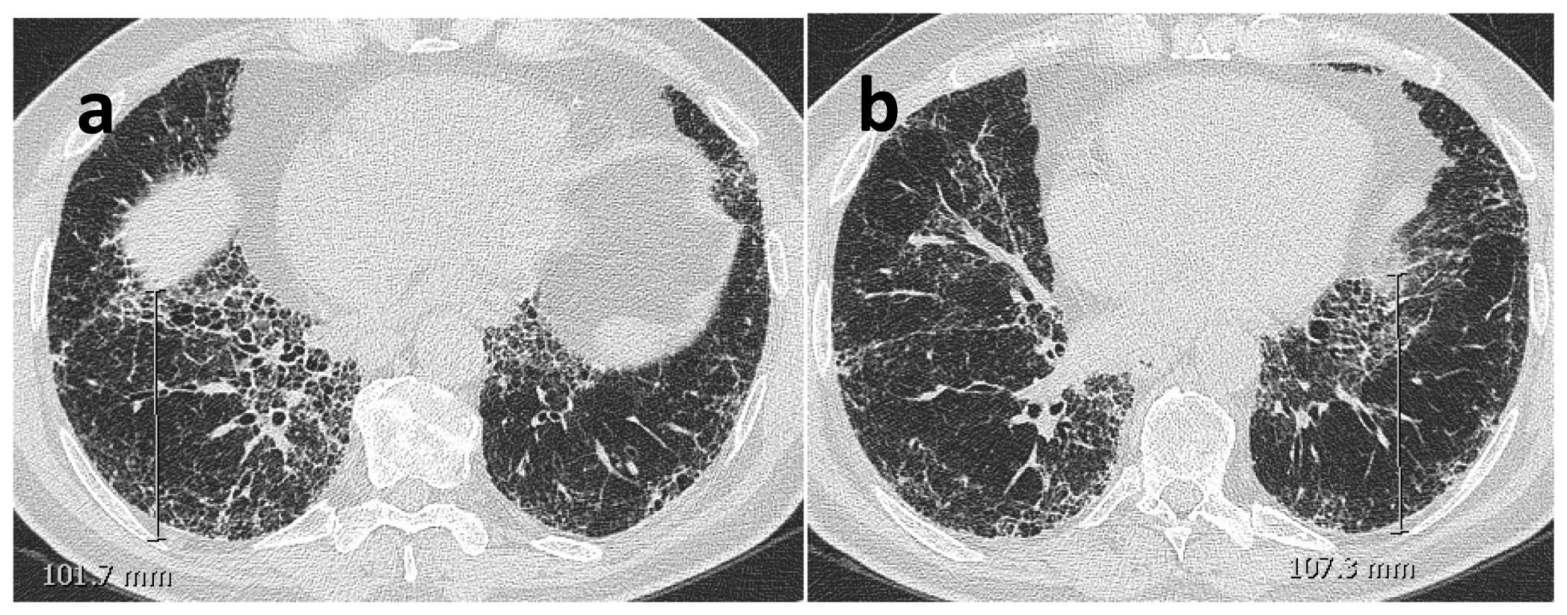
Axial non-contrast enhanced CT images of a 71-year-old male with IPF. The distance between where the fissure meets the diaphragm to the most posterior aspect of the lung was measured on the right (a). The measurement was repeated on the left lung (b) and mean value was calculated to obtain the oblique fissure posterior retraction distance (OFPRD).

**Figure 4:**
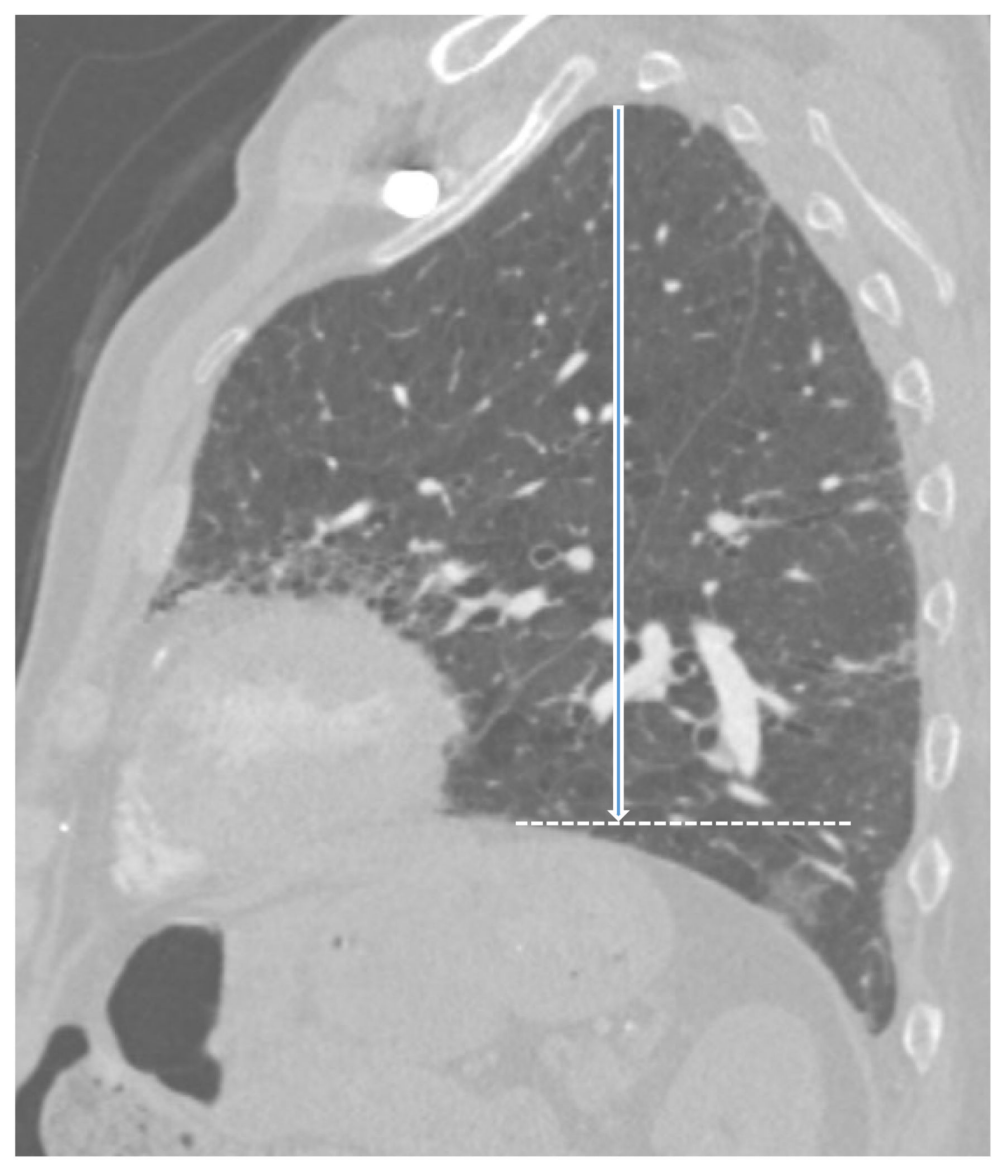
Sagittal contrast enhanced CT image of an 83-year-old male with IPF. CT image shows lung height (white arrow), drawn from lung apex to the dome of diaphragm (dashed line) at midclavicular line. Lung height was corrected for body habitus by dividing it by sagittal height of the tallest vertebrae in lower third of thoracic spine (not shown).

Previously described and reproducible CT parameters of regional volume loss (22,23) were evaluated at baseline and follow up CT by one experienced thoracic radiologist blinded to the chronology of CT scans. The measured variables were recorded in mm and were as follows:

1) The Aortosternal Distance (ASD) which is the distance between the anterior aspect of the ascending thoracic aorta and the retrosternum on axial slices. The pulmonary trunk was used as the anatomical landmark for standardising the HRCT slice (Figure 2).
2) The Oblique Fissure Posterior Retraction Distance (OFPRD) which is derived from measuring from the point that the fissure meets the diaphragm to the most posterior aspect of the lung on both sides on axial slices. The results are then averaged between the two lungs (Figure 3).
3) The Lung Height corrected for body habitus (LH) which is the distance between the lung apex and the diaphragmatic dome on sagittal reformats. The slice selection was standardised by using the midclavicular line (where the clavicle is seen midpoint alongside the first rib). Considering that lung height is related to an individual’s height, the measurement was corrected for body habitus by dividing it by sagittal height of the tallest vertebrae in the lower third of the thoracic spine (Figure 4).

### Semi-quantitative assessment of serial change in lung fibrosis extent (Fibrosis progression score)

To assess fibrosis progression on CT, one observer (12 years experience in thoracic radiology) simultaneously compared serial CTs obtained at two time points, blinded to the chronology of the scans. Fibrosis progression was categorized as: 0= no progression, 1= equivocal or minimal progression and 2= unequivocal progression. Unequivocal progression was defined as definite and visually perceptible progression in honeycombing or reticular and groundglass opacities associated with volume loss or traction bronchiectasis. Equivocal progression was used to describe cases where the identification of increased areas of reticulation or ground-glass opacification was uncertain.

To mitigate the potential impact of emphysema on lung volumes, baseline CTs were independently scored for emphysema extent by two experienced chest radiologists (5 and 7 years of experience respectively) on a lobar basis to the nearest 5%. The averaged values between the two raters were used as final emphysema score except for discrepant cases. Cases with discrepant readings between observers were identified by plotting the spread of differences between observers. The most disparate 5% (2 standard deviations) of values were adjudicated by a third experienced chest radiologist (7 years of experience). Emphysema scores at baseline were then reviewed with the intension of excluding those with emphysema scores ≥ 15 % from any analyses involving changes in FVC based on the findings of Cottin et al. (24).

### Pulmonary Function Tests (PFT)

FVC was expressed in liters and as % predicted values with DLCO (diffusing lung capacity for carbon monoxide) and FEV1 (forced expiratory volume) being expressed as % predicted values using the patient’s age, sex, race and height based on the recommendations of the European Coal and Steel Community (25). To account for baseline disease severity, the composite physiological index (CPI) was calculated using percentage predicted values for FEV1, FVC and DLCO (26). TLC was expressed in liters, using standard spirometry and plethysmography equipment.

### Statistical methods

Kolmogorov-Smirnov test was used to test for normality of the data. Data are expressed as mean and standard deviation, median with range or as number of patients and percentage as appropriate. The absolute difference between baseline and follow up values of CT measurements of regional volume loss (measured in mm), FVC (liters) and CT-derived automated lung volumes (liters) was calculated as:

$$Absolute\;difference\;={\;\text{Variable}}_{\text{follow-up}}\;-{\;\text{Variable}}_{\text{baseline}}$$

The percentage change in the variable in question from baseline to the follow up investigation was derived from the equation below:

Percentagechange=(Absolutedifference/variableatbaseline)×100

The annual percentage change in the variable in question from baseline to the follow up investigation was calculated as:

$$\begin{array}{l} Annual\;percentage\;change\;\text{=}\;\text{Percentage}\;\text{change}\;\text{x}\;\text{12/Time}\;\text{interval}\;\text{between}\;\text{baseline}\;\text{and}\\ \text{follow-up}\;\text{investigation}\;\text{(months)} \end{array}$$

The relationship between annual percentage change in CT measurements of regional volume loss and FVC was evaluated using linear regression analysis.

To assess the value of CT measurements of regional volume loss in determining outcome in IPF, survival analysis against mortality was carried out for each CT variable using Cox proportional hazard analysis that included age, gender and baseline CPI to correct for baseline disease severity. The strongest predictor of outcome amongst CT markers of volume loss was then included in a confirmatory multivariable survival analysis that also included established prognostic markers in IPF, namely serial change in FVC and CT fibrosis progression score.

To establish a clinically applicable threshold for CT parameters of volumes loss as a predictor of outcome in IPF, a histogram analysis was performed to identify the optimal cut-off to subsequently test in a multivariate Cox proportional hazard analysis which included FVC decline ≥ 10% as well as fibrosis progression. P values less than 0.05 was regarded as statistically significant.

Statistical analyses were performed with SPSS (IBM Corp. Released 2016. IBM SPSS Statistics for Windows, Version 24.0. Armonk, NY: IBM Corp).

## Results

### Study population

The final population comprised 81 patients with concurrent sequential CT and PFT. There were 58 deaths during the study period (n=58/81, 72%) with median survival time of 17 months (range 0.3-57 months). Age, gender, PFT data, and CT measurements are shown in Table 1.

23 patients (n=23/81, 28%) had accompanying emphysema with only 5 patients (n=5/81, 6%) having emphysema scores ≥ 15% (range 15-51%). On this basis, these patients were not excluded from the overall analysis. The majority of patients showed no evidence of progressive fibrosis on serial CT (n=57/81, 70%); 19 patients (n=19/81, 23%) showed unequivocal progression and 5 patients showed equivocal progression (n=5/81,6%). No patients showed reduction in the extent of fibrosis on follow-up CT.

### Adequacy of CTs for analysis

All patients had successful automated lung volume (ALV) measurement at baseline. On follow-up imaging, ALV calculation was feasible in 72 patients (72/81, 89%) but failed in 9 patients on (n=9/81, 11%) due to inability of the software to segment the lungs. OFPRD was not recorded in 2 patients at baseline (2/81, 2.5%) and in 1 patient at follow-up (1/81, 1%) because the severely distorted fissures could not be accurately identified. ASD was recorded in all patients at baseline and not recorded in 1 patient at follow-up (1/81, 1%), due to interim coronary artery bypass graft surgery and the resultant distortion of the mediastinum. LH was recorded in all cases at both time points.

### The relationship between serial change in CT measurements of volume loss and serial change in FVC

Annual% change in ALV was significantly related to annual% change in FVC (R^2^ = 0.26, P<0.0001). As shown in Table 2, annual% change in ASD, LH and OFPRD were predictors of annual% change in FVC, with OFPRD being the strongest determinant.

Multiple stepwise linear regression analysis was conducted to ascertain if annual change in FVC could be predicted based on a combination of annual change in morphologic variables and ALV. The change in ALV was the only determinant of the change in FVC with model R^2^ of 0.27 (ß=0.63, P<0.001). However, the annual% changes in the morphologic CT variables were not determinant of change in FVC.

### Independent Relationship between CT measurements of volume loss and outcome

As shown in Table 3, annual percentage change in ASD was the strongest predictor of mortality when corrected for baseline disease severity, age and gender (HR=0.97, P=0.001). Ensuing multivariate survival analysis combining annual percentage change in ASD, FVC and fibrosis progression showed that annual percentage change in ASD was the only remaining variable predicting mortality (HR=0.97, P<0.001) (Table 4).

**Figure 5:**
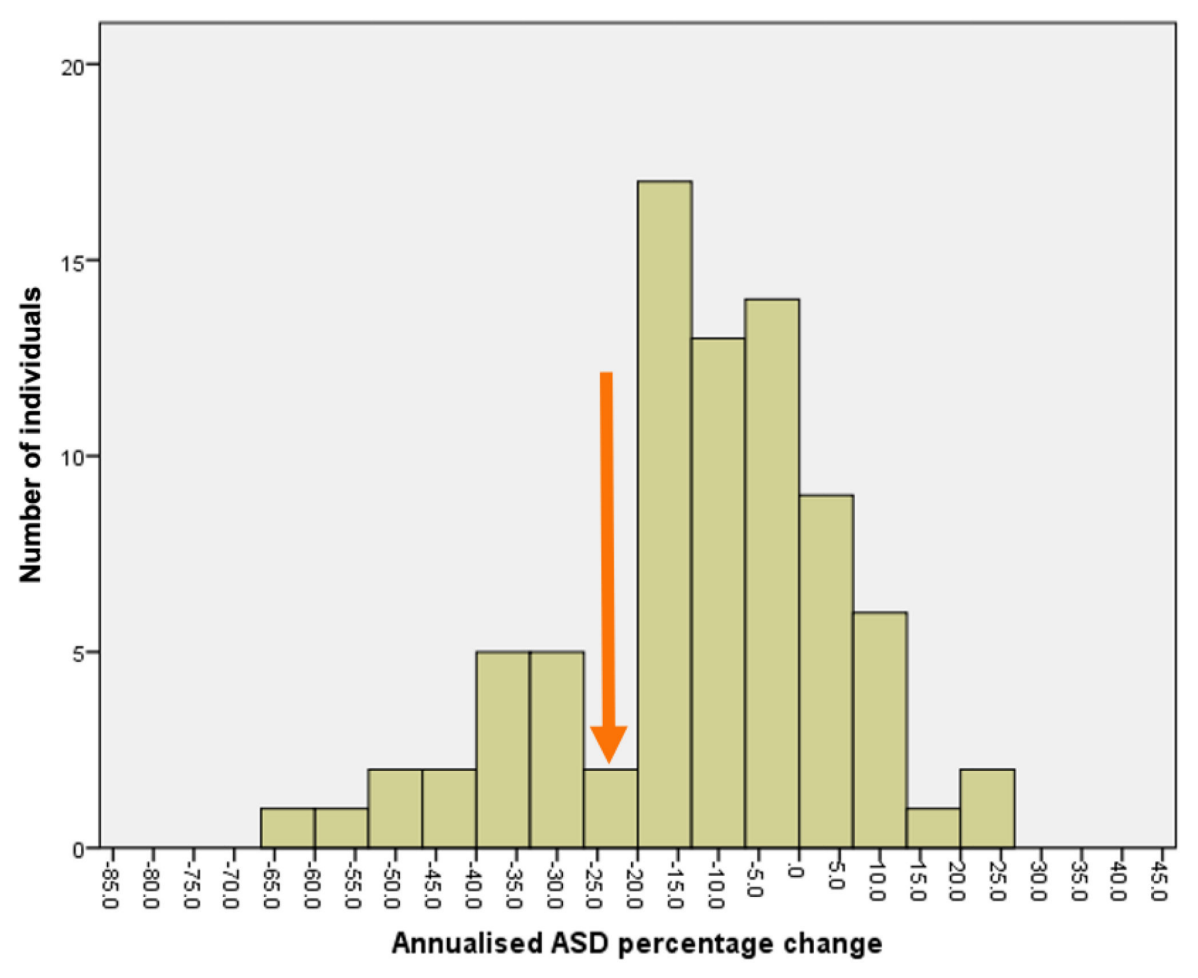
Histogram analysis of annualised change in ASD plots (Ann ASD perc change) in 5% sections showing a bimodal distribution with nadir at approximately 25% (orange arrow).

**Figure 6:**
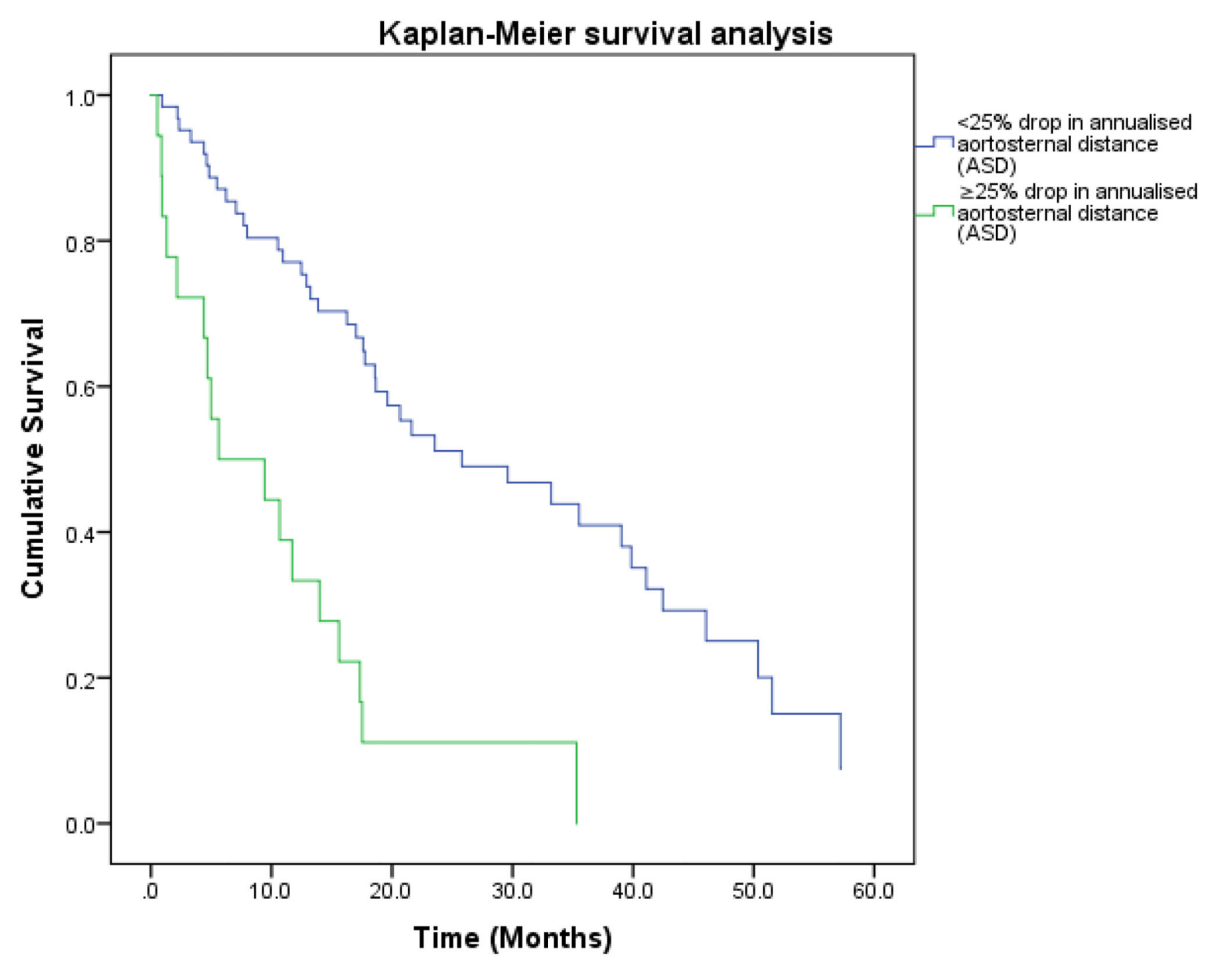
Survival curves for patients with idiopathic pulmonary fibrosis separated into two groups according to presence or absence of 25% drop in annualised aortosternal distance (ASD). The curves confirm significant survival difference between the two groups (P<0.001).

A histogram analysis of the annual percentage change in ASD data was performed (Figure 5). The histogram was plotted in 5% sections and showed a bimodal frequency distribution with a nadir at approximately 25%. When used as a binary variable in multivariate analysis with FVC decline of ≥ 10% and CT fibrosis progression, only the presence of a 25% reduction in annualised ASD remained predictive of outcome. (HR=3.8, p<0.001) (Table 5), illustrated in Kaplan-Meier (Figure 6).

## Discussion

Assessing disease severity and progression in patients with IPF is of paramount importance because it allows clinicians to decide on initiating treatment, and to assess the impact of treatment, whilst simultaneously providing prognostic information. In this study, we tested the ability of serial CT measurements of lung volume and volume loss to act as markers of disease progression and found that reduction in the aortosternal distance (ASD), (i.e. the distance between the retrosternum and anterior aspect of the ascending thoracic aorta) was the strongest predictor of mortality, outperforming the annual relative percentage change in FVC. Furthermore, we demonstrated that a decline of 25% in ASD predicts mortality superior to a decline in FVC of 10% or more.

This finding is relevant because pulmonary function test parameters, namely FVC is currently the most widely accepted method of assessing disease progression and response to treatment in IPF. However, these parameters are subject to inter and intra-test variability, are difficult to interpret when there is marginal change (5-10%) and are commonly influenced by co-existing emphysema and/or pulmonary hypertension (7,27). From a research standpoint, studies require novel sensitive markers that can capture change in disease severity over time, particularly given the role of antifibrotic agents in delaying FVC decline in IPF. Consequently, there is mounting interest to develop new markers of disease progression in IPF. In this context, the prospect of developing easily measurable markers of lung volume loss as a measure of disease progression derived from routine imaging is attractive.

The reason for the superior signal provided by serial change in ASD in predicting mortality when compared with other measures of volume loss on CT that we tested is not immediately clear. The ASD is primarily an upper zone measurement while IPF is a lower lobe predominant disease. It may be that increasing fibrosis and volume loss in progressive IPF causes a reduction in anteroposterior thoracic anatomical distances regardless of zonal dominance.

We found that while serial change in automated CT derived lung volume closely correlated with change in FVC, it was a weaker predictor of mortality when compared to the ASD. This suggests that reductions in ASD over time may reflect more clinically significant changes in pathophysiology than overall lung volumes in IPF.

It is notable that in our data, visually assessed fibrosis progression was not an independent predictor of mortality in any of the survival analyses. This highlights the lack of consensus about the use and significance of subjectively comparing fibrosis on serial CT. There are previous studies concluding that fibrosis progression on CT is an indicator of poor outcomes (8,9,11,28). On the other hand, there are also reports indicating that progressive fibrosis on serial imaging is not as strong predictor of mortality as the extent of fibrosis at baseline (8,29). The reason for the disparate conclusions could be down to differences in population characteristics and methods of assessing disease progression. In our cohort, there was a small number of patients with definite progressive fibrosis, possibly due to more severe baseline disease. To evaluate disease progression, we used the more clinically applicable three scale method as opposed to the formal scoring which gives an estimate of the percentage of the lung affected by a particular parenchymal abnormality that may be more sensitive in capturing progressive disease.

The present study has some limitations. There was no spirometric gating of the CT scans in this study, which could mean that there was a variable state of inflation of the lungs, thus altering the position of the diaphragm and fissures. However, spirometric gating is not widely used in clinical practice and addressing this limitation would likely only further strengthen the relationships that we have found. Also, the data was collected retrospectively comprising of clinically indicated CT examinations which may result in bias towards including patients with more likelihood of disease progression, and therefore it is not certain whether these results apply to patients with more limited disease. It is also acknowledged that the study is from a single centre, and it is important that the results are validated externally before they can be used in clinical practice. Moreover, further work is required to establish the minimal meaningful change in ASD, that denotes clinically significant progression in IPF (2,30,31). Nevertheless, the results of this study could potentially direct further work on automated techniques in fibrotic lung disease severity assessment which currently has concentrated primarily on the extent of parenchymal abnormalities such as reticulation, honeycombing and ground glass opacity only, rather than lung volumes.

In conclusion, we have demonstrated that serial changes in automated and surrogate CT markers of volume loss correspond with changes in FVC over time and specifically that 25% reduction in the aortosternal distance can provide evidence of disease progression beyond that identified by FVC trends, validated against mortality.

## Figures and Tables

**Table 1:** Patients Demographics and pulmonary function and CT Data. Data expressed as mean +/- standard deviation or as median (range) as appropriate.

Variable (n = 81 Unless Stated)	Value
Age Median (y)	67
Age Range (y)	38 – 85
Male/Female Ratio	66/15 (81%/19%)
Baseline FVC% predicted	72±21
Baseline FEV1% predicted(n=80)	74±19
Baseline DLCO% predicted	39±13
Baseline CPI (n=80)	53±12.5
Baseline TLC% predicted (n=74)	65±18
Follow-up FVC% predicted	63±23
Annual %change in FVC (L)	-12 (-107, 23)
Annual %change in DLCO (n=75)	-22±20
Emphysema present	n=23, 28%
Emphysema Score%	0 (0,51)
Annual % change in ALV (n = 72)	-6 (-68.5, 39)
Annual %change in LH	-2.2±7.5
Annual %change in ASD (n=80)	-10 (-65,25)
Annual %change in OFPRD (n=78)	-5.6 (-39,24)

Data represent mean values with SDs or median values with range as appropriate. FVC, FEV1, DLCO and TLC represent forced vital capacity, forced expiratory volume in 1 second, diffusion capacity for carbon monoxide and total lung capacity respectively. CPI stands for composite physiological index. ALV, LH,ASD and OFPRD represent automated total lung volume, lung height corrected for body habitus, aortosternal distance and oblique fissure posterior retraction distance.

**Table 2:** Univariate regression analyses demonstrating relationships between annual percentage change in CT variables and FVC.

PFT	CT variable	β Coefficient	95% Confidence Interval	*P* Value	Model *R*^2^
Annual%	OFPRD	0.86	0.37-1.34	0.001	0.140
Change	ASD	0.28	0.03-0.53	0.025	0.060
in FVC	LH	0.87	0.31-1.42	0.003	0.003

PFT =Pulmonary function test. FVC represents forced vital capacity. OFPRD represents oblique fissure posterior retraction distance. ASD represents aortosternal distance. LH represents sagittal lung height corrected for body habitus.

**Table 3:** Multivariate regression analysis demonstrating mortality according to annual percentage change in CT variables, age, baseline CPI and gender (n=81).

Models	Variables in the model	HR	*P* Value
1	OFPRD	0.95	0.002
	Baseline CPI	1.05	<0.001
	Age	0.99	0.800
	Gender	1.20	0.600
2	ASD	0.97	0.001
	Baseline CPI	1.05	<0.001
	Age	0.98	0.300
	Gender	1.00	0.100
3	LH	0.96	0.040
	Baseline CPI	1.05	<0.001
	Age	0.99	0.900
	Gender	0.97	0.940
4	ALV	0.98	0.021
	Baseline CPI	1.05	0.001
	Age	0.99	0.600
	Gender	1.26	0.600
5	Fibrosis progression	1.03	0.800
	Baseline CPI	1.05	<0.001
	Age	0.99	0.700
	Gender	1.01	0.100

OFPRD, ASD & LH represent oblique fissure posterior retraction distance, aortosternal distance and lung height corrected for body habitus respectively. ALV represents automated total lung volume. CPI represents composite physiological index.

**Table 4:** Multivariate regression analysis demonstrating mortality according to annual percentage change in ASD, FVC and CT fibrosis progression (n=81).

CT Variable	HR	*P* Value
ASD	0.97	<0.001
FVC	0.98	0.070
Fibrosis progression	1.03	0.840

ASD represents aortosternal distance. FVC represents forced vital capacity.

**Table 5:** Multivariate regression analysis demonstrating mortality according to 25% annualised drop in ASD, 10% decline in FVC and CT fibrosis progression (n=81).

CT Variable	HR	P Vaue
Presence of 25% drop in annualised ASD	3.80	<0.001
FVC drop ≥ 10%	1.34	0.30
Fibrosis progression	1.02	0.88

ASD represents aortosternal distance. FVC represents forced vital capacity.

## Key Points

1. Serial decline in automated and surrogate markers of lung volume on CT corresponds to changes in FVC.
2. Annualised reductions in the distance between ascending aorta and retrosternum on CT predicts mortality beyond annualised percentage change in FVC.
